# Supplementary material for: Network Pharmacology-Based Characterization of Mecasin (KCHO-1) as a Multi-Target Modulator of Neuroinflammatory Pathways in Alzheimer’s Disease
Source: Nutrients. 2025 Dec 19;18(1):8. doi: 10.3390/nu18010008 (PMC12787420; doi:10.3390/nu18010008)
Supplement: Supplementary file 1 [file nutrients-18-00008-s001.zip › nutrients-4011336-supplementary.pdf]

Supplementary Table S1. List of overlapping target genes shared between Mecasin and Alzheimer's disease gene sets.

942 Common Genes of Mecasin and Alzheimer's Disease
PTGS2, ACHE, BCL2, MAPK8, FOS, TNF, DNAH9, MAPK14, CASP3, GAA, TYR, AKT1, IL6, NFE2L2, ANXA5, F2, IL2, PIK3CA, NKRF, BIRC2, MAPK7, ELAVL1, CD83, TYRP1, TNFRSF10A, CD80, GBA1, NOTCH1, CTSB, CYP2E1, BIRC5, AHR, CD40, CASP1, BCL2L1, PTGS1, PRKAA2, NLRP3, GSR, CASP9, MKI67, TLR4, HMOX1, STAT3, CTNNB1, BDNF, POMC, EGF, CXCL8, CYP1B1, MTOR, EGFR, IFNLR1, VEGFA, CD4, INS, VCAM1, TLR1, NFKBIA, KEAP1, SREBF1, TLR2, PPARA, GABPA, ANG, GPT, CRP, TP53, JUND, CAT, NFKB1, RELA, CCND1, IL10, ABCB1, CCL2, CDKN1A, NOS2, PARP1, PPARG,, MPO, CASP8, ALB, CD44, MYC, CCK, SIRT1, CDH1, EP300, PTEN, IL17A, PIK3C3, SNCA, VIM, IL4, HIF1A, ERBB2, NQO1, GFAP, PCNA, JAK2, TNK1, MIR21, MAPK1, IL1B, ITGAM, DDIT3, CYP3A4, GSTA4, SERPINB1, GSTA1, FPR2, CASR, MME, CXCR4, SPP1, MAPK3, CYP2C9, CYP2C19, CYP2D6, CYP1A2, ARG1, ELANE, ESR1, PRPF19, CYP2C8, CYP2A6, MLN, VIP, CHAT, GAST, TAC1, SST, CRTC1, RB1, RBL2, GAP43, NTRK2, NGF, ARAF, CA1, BCHE, BECN1, MAP1LC3A, ADORA2A, TH, BACE1, GRIA1, DCTN4, NFKBIB, SOD2, MATK, FDFT1, CHUK, CD69, ATG5, MAPK9, ABCC2, NTRK1, MYD88, GRIN2B, IL13, ICAM1, NR3C1, ATP2B1, GCG, HSP90B1, LEP, LYZ, NT5C2, ADIPOQ, SLC5A1, LIPE, CD79A, GCK, TNR, G6PD, TF, PKM, AKR1B1, ATRN, SLC2A2, INSR, SLC2A4, SMARCA4, ST3GAL4, SLC2A1, DLAT, HTT, ROCK2, SERPINF1, RHOA, SOD1, HIPK2, RAC2, AKR1A1, OMP, DNMT1, MARK2, DPP4, BMP3, PON1, CALR, PLA2G7, FH, SLC38A5, RRS1, PON3, PON2, ATF1, ADA, TXNIP, FASN, HMGB1, APOA1, TXN, TM4SF1, SRR, APOB, MMUT, ATF2, ME2, CTRL, LCAT, CS, OGT, MGAT3, MGAT5, GNPTAB, NAGK, FUT8, GFPT1, SELE, NAGA, B4GALT1, GNPTG, SP1, HPSE, CTSA, MBL2, LGALS3, NDST1, HMGA2, CTSD, SLC3A2, GALNS, XDH, RUNX2, IL18, NOS3, KITLG, LPL, CD36, ACE2, BMP2, TNFSF11, AGTR1, XIAP, MAP3K14, GRB2, ACE, CREBBP, GRM1, ARTN, CYBB, GJA1, NR3C2, CRH, EIF2AK3, NT5C3A, MX1, FOSB, PRNP, CAMKK2, CREB1, CA2, MMP9, STAT1, CDK2, ABCG2, PLA2G12A, PTH, TMEM115, DPP6, RBP1, TXNRD3, CBX4, CDK4, IDO1, PTK2, AIMP2, PRTN3, RPS2, ZNF346, ZMYND8, PRKN, CEP104, HMGCR, DEGS1, BAK1, ABI1, ABAT, DBH, COASY, PDS5B, LOX, AOC3, SYK, KCNA3, NASP, C1ORF87, XAB2, CUL2, CYP1A1, PFDN5, AXIN2, IL1RN, PNN, SLC38A3, CKAP2, CYP2B6, TACR1, SULT1B1, PRKDC, COMT, XRCC5, ANK1, TBXA2R, YAP1, PML, PECAM1, HGF, NPY, EDN1, DNTT, PIP, PARK7, AGER, HDAC2, NOX4, RBFOX3, KDR, APOE, GGPS1, CPS1, AURKA, NR1I2, AR, CDKN1B, PCSK9, CDKN2A, ISG15, PRDX1, ABCA1, EIF2A, NCOA2, ZBP1, IKBKB, ITGA2, PTPN1, CCNB1, MMP2, MCL1, PTPN11, SMAD3, SRARP, GCLC, ERN1, GPER1, PTPN6, JUN, MAP2K1, TNFRSF10B, CCN2, SRC, PRKCD, HSPA5, TGFB1, GSDMD, TAT, CCL11, SERPINH1, PPARGC1A, C3, TJP1, TAGLN2, SELP, CYLD, CRYAB, EPHA2, SLC22A8, APAF1, NRF1, TFAM, CLDN5, CDK6, ABCC3, EIF2AK2, CAV1, SMAD2, P2RY12, THBD, TNFRSF11B, BGLAP, PDIA3, S100A4, MMP13, TNNI3, SLC7A11, MAOA, COL1A1, GPX4, SERPINC1, DRD1, TAF9, APOA2, LGALS1, TKT, TOP1, CD14, ISG20, ALOX5, MGMT, TMEM123, PSMC5, PRKCB, NFE2, CDH5, P2RX7, SOX2, CYP27A1, DAO, OTC, CHRNA4, SLC16A1, CPE, GNRH1, CHRNA7, MB, RARA, MBP, PRL, FBXO7, FDXR, SLC22A5, CRAT, CPT2, SLC25A20, SLC22A2, GCDH, SLC22A1, EPO, CPT1B, HADHA, HADH, TMLHE, SLC22A3, SLC25A13, ALDH9A1, CPT1C, CBLC, MMACHC, CROT, ACAD8, IGF1, FMO3, UCP3, DMD, SLCO2B1, FGF21, VKORC1, MAOB, PROS1, CA9, F7, TUBB4B, CYP19A1, CYP3A5, PF4, ERH, REN, DLST, CALCA, CSF3, KNG1, FECH, CPOX, HMBS, UROD, HPX, TSPO, TFRC, HMOX2,

BFSP1, HP, FTH1, ALAS1, FXN, CP, BACH1, IREB2, TPH2, SLC6A4, KYNU, WARS1, KMO, MYLK, XRCC1, PAH, HTR1A, IFNG, RHO, ADRB3, AADAT, HTR2A, FDPS, YWHAZ, ERVW-1, NOS1, PRMT1, PRMT5, ARG2, GHRH, ASS1, AZIN2, ODC1, PADI4, CARM1, C5AR1, SLC7A1, PLG, SRSF1, SRPK1, AVP, FURIN, GATM, OAT, SLC10A2, NR1H4,SLCO1A2,SLCO1C1, SCT, SLC51A, ABCC4, CYP8B1, ABCC1, ABCG5, S1PR2, NR0B2, GPBAR1, CEL, SLC10A4, S100A12, CXCL2, REG3A, ENTPD2, GH1, VAT1, CLPP, ITPR3, USP47, DVL1, DVL2, FZD2, MAP3K12, OPRD1, OPRM1, TMEM97, FAAH, PDYN, CDCA4, SF3B1, C3AR1, KIR3DL1, FFAR3, CACNA1B, PIK3CG, DYRK1A, SIRT2, ENO1, OPRL1, DLK1, CACNA1D, DUOX2, NOX1, RPS6, CACNA1A, PTGER4, HTR2C, ADRA1A, HTR3A, CCR5, TPM3, RAC1, DRD2, BAAT, LCT, TOR1A, NBR1, CLDN1, B2M, GLP1R, NTS, GSK3A, ZEB2, SLC29A4, CD68, KCNB1, CXCR3, FAS, FADD, FASLG, BIRC3, SOCS3, F3, CCL5, TLR9, ELN, CXCL1, RPS28, ACYP2, GSTM4, PRMT2, UBE2T, DLGAP5, B3GLCT, TNFAIP6, GSE1, KLF15, POR, MCOLN1, WRN, ACO2, HFE, ACO1, FN1, TFEB, LAMP1, CCL4, PTGES, RPS6KB1, LPO, CDC42, FOXO1, GAPDH, ESR2, NOTCH2, SULT1A3, UCP1, GRK2, ADORA1, IL23A, STAT6, PIK3R1, NEUROD2, CHMP2A, NEUROG1, STMN4, ESAM, HRH1, SEMA3A, PLCG1, TSLP, TGFBR1, PIK3CD, PYCARD, NTF3, PDK1, CEBPA, GAD1, PTGER2, SIRT3, CTSK, HDAC9, AIF1, NPPB, CD1A, NRG1, TXNRD1, CXCL12, NAAA, CCND3, NFATC1, VWF, UPP2, DAD1, IGFBP6, HBD, CD200R1, IRAK1, TRAF6, ICAM5, CCR2, SGCB, RAF1, MDM2, PC, ACAT1, UCP2, MARCKS, PANK2, KCNN4, MFN2, MGLL, MAPK10, PLAU, CADM1, IL3, OPA1, FGF2, CSF2, SPRY2, MFN1, OLR1, FGF1, SYN1, VHL, KAT5, AP2M1, CSF1, C1QA, GJA5, HSD11B1, ZBTB10, SP3, ALDH2, ALDH1A1, JAK1, CASP7, DESI1, DESI2, PGR, CDR1, GLA, AKT2, CBLB, TTR, NUFIP2, DECR1, TCIRG1, HAX1, LMNA, CNDP1, CNDP2, MGP, NR1H3, TRIM33, ADRB2, CDK1, TMPRSS2, CCL7, EPCAM, IL6R, IRS1, RPE, ARNT, MAP2, SYP, SQSTM1, TNFRSF11A, GHRL, CDH2, ULK1, XBP1, PFKFB3, PER1, PER2, ESRRB, SH3GL1, IGF1R, HSP90AA1, CSNK2A1, CNP, PTK2B, CFTR, PXN, ITGB1, DUSP1, TFAP4, CFH, RXRA, CDK5, PRF1, IAPP, SHBG, BRCA1, KLK3, FADS1, OSM, FADS2, DLG4, MMP7, CD274, PDCD1, LRP5, DKK1, KIF11, PLIN1, NR1H2, ENO3, CALU, GSK3B, CASP4, PRKCA, UBB, NEK7, BDKRB2, CLTB, CDC25B, NLRC4, CDC25A, GATA3, ATF3, ERBB3, CNR2, AIFM1, HSPB8, MMP1, IL2RA, GGA3, GGA1, PGF, IL15, MATR3, DNAJB7, BCL2L2, HTR1B, MAP3K11, SNAI2, CLDN3, MEFV, MET, OCLN, ICMT, SOAT1, PTPRC, GLO1, SYT1, AKR1B10, SORD, ATF6, THRB, SP4, LCN2, PTCH1, LARP1, SREBF2, SCAP, PAX3, ACAN, RPS6KA1, RPS6KA2, SCARB1, ABCG1, MFGE8, CPT1A, CXCL10, DUSP3, SENP1, NOP2, SERPINF2, EIF4EBP1, HGS, CGAS, CSNK2A2, KCNQ2, KCNQ1, FPR1, GABRB2, GABRA5, GABRG2, C1ORF116, SRPX2, MMP12, POLD3, IGF2R, TPH1, CYP51A1, POLB, CXCL9, OGG1, RYR2, KCNJ5, PCSK5, SLC8A1, RYR1, CAMK2G, BNIP3, PSEN1, CAMK2A, BMAL1, CNR1, ADAMTS9, SOX5, ADAMTS4, ADAMTS5, HDAC3, LDHA, CYSLTR2, AQP1, INA, GSTK1, AGT, CD28, SLC5A2, RETN, HBA1, FGFR1, COL4A2, NOTCH4, SLC38A1, NFATC2, SLC1A5, ADIPOR2, ADIPOR1, JAK3, FGFR2, SPHK1, SOX9, GLS, ATF4, FBP1, PPIB, PFKP, TIGAR, GLUL, ALDOA, INSIG1, GHSR, NPM1, CD2AP, AMOTL2, KDM4A, SYNPO, KDM6A, CAMK4, JAG1, TRPC6, KL, SCD, VDAC1, ARHGAP45
